# Genome comparisons provide insights into the role of secondary metabolites in the pathogenic phase of the *Photorhabdus* life cycle

**DOI:** 10.1186/s12864-016-2862-4

**Published:** 2016-08-03

**Authors:** Nicholas J. Tobias, Bagdevi Mishra, Deepak K. Gupta, Rahul Sharma, Marco Thines, Timothy P. Stinear, Helge B. Bode

**Affiliations:** 1 Fachbereich Biowissenschaften, Merck Stiftungsprofessur für Molekulare Biotechnologie, Goethe Universität Frankfurt, Frankfurt am Main, Germany; 2 Biodiversity and Climate Research Centre (BiK-F), Senckenberg Gesellschaft für Naturforschung, Senckenberganlage 25, 60325 Frankfurt am Main, Germany; 3 Fachbereich Biowissenschaften, Institut für Ökologie, Evolution und Diversität, Goethe Universität Frankfurt, Max-von-Laue-Str. 13, 60438 Frankfurt am Main, Germany; 4 Department of Microbiology and Immunology, University of Melbourne, at the Doherty Institute for Infection and Immunity, Parkville, VIC 3010 Australia; 5 Buchmann Institute for Molecular Life Sciences (BMLS), Goethe Universität Frankfurt, Frankfurt am Main, Germany

**Keywords:** *Photorhabdus*, Sequencing, Secondary metabolites, Symbiosis

## Abstract

**Background:**

Bacteria within the genus *Photorhabdus* maintain mutualistic symbioses with nematodes in complicated lifecycles that also involves insect pathogenic phases. Intriguingly, these bacteria are rich in biosynthetic gene clusters that produce compounds with diverse biological activities. As a basis to better understand the life cycles of *Photorhabdus* we sequenced the genomes of two recently discovered representative species and performed detailed genomic comparisons with five publically available genomes.

**Results:**

Here we report the genomic details of two new reference *Photorhabdus* species. By then conducting genomic comparisons across the genus, we show that there are several highly conserved biosynthetic gene clusters. These clusters produce a range of bioactive small molecules that support the pathogenic phase of the integral relationship that *Photorhabdus* maintain with nematodes.

**Conclusions:**

*Photorhabdus* contain several genetic loci that allow them to become specialist insect pathogens by efficiently evading insect immune responses and killing the insect host.

**Electronic supplementary material:**

The online version of this article (doi:10.1186/s12864-016-2862-4) contains supplementary material, which is available to authorized users.

## Background

Members of the genus *Photorhabdus* include both insect and human pathogens. Despite only three distinct species described to date (*P. luminescens, P. temperata* and *P. asymbiotica*), significant sequence divergence within each species has led to the identification of several subspecies [1–7]. All three species maintain complex life cycles that include a nematode mutualistic symbiont as well as a pathogenic phase. During the symbiotic phase, the bacteria colonize nematodes of the genus *Heterorhabditis* during the infective juvenile (IJ) stage. The nematodes are generally free living in soil and seek out insects to infect so as to utilize the nutrients for growth and perpetuation of their progeny [8]. This is the dominant life cycle of the *Photorhabdus* however, occasional human infections by *P. asymbiotica* do occur [9]. During the infective stage, nematodes enter the insect and release the bacteria directly into the hemolymph where the bacteria also proliferate and eventually kill the insect. The insect cadaver provides a rich source of nutrients for both the nematode and the bacteria. Following proliferation of both, the bacteria re-colonize the nematode IJs before re-entering the soil in search of a new host [8].

Throughout this existence, the nematodes provide the bacteria with a means of transport while the bacteria supply a variety of secondary metabolites produced by biosynthetic gene clusters (BGCs). Products of these BGCs are small molecules, frequently polyketides (PK), or non-ribosomal peptides (NRP) and can additionally include bacteriocins, siderophores and fatty acids among others. While there are common themes in their biosynthesis, each class of small molecule has a different mechanism of production and probably varying functions, with the majority of currently known metabolites reported as having some antimicrobial role [10–16]. Not all of these metabolites are required for symbiosis [17] so secondary metabolite biosynthesis alone - while important - does not explain the conservation of their corresponding genetic loci among closely related *Photorhabdus* or other entomopathogens of the genus *Xenorhabdus* [18].

The conservation of these general types of molecules led us to investigate whether there was a more generally conserved function. Through genome mining and using representative genomes from each species (and subspecies) of *Photorhabdus*, we compare seven different genomes in order to better understand the differences between the specific niche of each bacterium and the key analogous functions among the shared protein-coding DNA sequences (CDS).

Significant research has been conducted on *Xenorhabdus* species and their response to infection of insects. The role of some compounds produced by members of both genera has firmly been established as symbiotic factors [17, 19, 20] while others are predicted to be involved in this process. A role for a small number of secondary metabolites has been proposed in nematode development, however the majority of the BGCs appear to have little effect on this process (unpublished data). Following insect infection by nematodes, the bacteria are released into the insect hemolymph, quickly activating the cellular and humoral immune responses against the causative pathogens via one of two pathways, the Toll or immunodeficiency (IMD) pathways. The Toll pathway is activated in response to infection by Gram-positive bacteria and fungi using pattern-recognition receptors that respond to pathogen-associated molecular patterns [21–23]. On the other hand, Gram-negative pathogens activate the IMD pathway. This differential activation results in expression of a distinct set of genes for each in response to the type of infection occurring. However, subsets of overlapping sequences that are activated in both pathways have been identified in *Drosophila* and act synergistically in order to more efficiently deal with invading organisms [24, 25]. Alternatively, prophenoloxidase (proPO) pathways can be activated by exposure to lipopolysaccharides, peptidoglycan, amphiphilic lipids or even damaged cells [26, 27]. ProPO is activated through cleavage by a serine protease resulting in active phenoloxidase (PO) that assists in pathogen isolation and lysis [28]. Several different serine protease inhibitors heavily regulate this system, as excess PO can be detrimental to the host [27, 29]. Some compounds from *P. luminescens* have recently been given defined roles in suppressing some parts of this insect immune response [30, 31].

One previous study has examined the similarities between *P. luminescens* and *Yersinia enterocolitica* in order to draw conclusions regarding key factors involved in insect pathogenesis [32]. In order to determine the conserved features of members of *Photorhabdus* and draw more specific conclusions with respect to the essential roles of proteins in the *Photorhabdus* lifecycle, we sequenced two novel isolates that, together with the already sequenced genomes, provide a broad geographical and genomic perspective of the genus. Using a comparative genomic approach, we highlight mechanisms that are conserved across the genus and predict possible functions of the products of the numerous BGCs and conserved signaling pathways.

## Results

### Genome composition of *Photorhabdus* spp. collected from Thailand

In order to establish a broad collection of *Photorhabdus* strains, we sequenced two additional isolates collected from Thailand [33]. However, Thanwisai et al. did note that the bacteria grouped into five distinct clades with Group 3 still lacking a reference strain. Sequencing of *Photorhabdus* PB45.5 and PB68.1 now provide reference sequences for Groups 3 and 5, respectively [33]. These Whole Genome Shotgun projects have been deposited at GenBank under the accession numbers LOIC00000000 and LOMY00000000, respectively.

Following sequencing and assembly (statistics available in Additional file 1), we performed an average nucleotide identity analysis on the genomes in order to determine the species. *Photorhabdus* PB68.1 was closely related to *P. asymbiotica* subsp. *australis* (ANI = 97.0 %) while *Photorhabdus* PB45.5 is most closely related to *P. luminescens* subsp. *laumondii* TTO1 (ANI = 91.4 %) and may represent a novel subspecies. The genomes consist of 4,918,001 and 5,425,505 bp with GC contents of 42.0 and 42.7 % respectively. *P. asymbiotica* PB68.1 is predicted to contain 4600 CDS whilst *Photorhabdus* PB45.5 contains only 4353 CDS.

Together with *P. luminescens* TTO1 (NC_005126) [2], *P. temperata* subsp. *khanii* NC19 (NZ_AYSJ00000000) [4, 34], *P. temperata* subsp. *temperata* M1021 (NZ_AUXQ00000000) [3], *P. temperata* subsp. *thracencis* DSM 15199 (NZ_CP011104) [1] and *P. asymbiotica* ATCC 43949 (NC_012962) [6] we identified ortholog families across the seven strains. During ortholog identification, all protein singletons were removed from further analysis. This analysis suggests that the core *Photorhabdus* genome consists of a total of 2101 CDS, 520 of which are absent in *E. coli* K12 (Additional file 2). Using the KAAS server [35], KEGG orthology numbers were assigned to the fully assembled genomes (Additional file 3) and mapping to KEGG pathways was performed (Additional file 4). No obvious differences were apparent except for a much greater number of two-component systems present in *P. luminescens* TTO1 (96) compared to either *P. asymbiotica* ATCC 43949 (87) or *P. temperata* subsp. *thracensis* DSM 15199 (84).

## Discussion

### Biosynthetic gene clusters are numerous and diverse

**Fig. 1:**
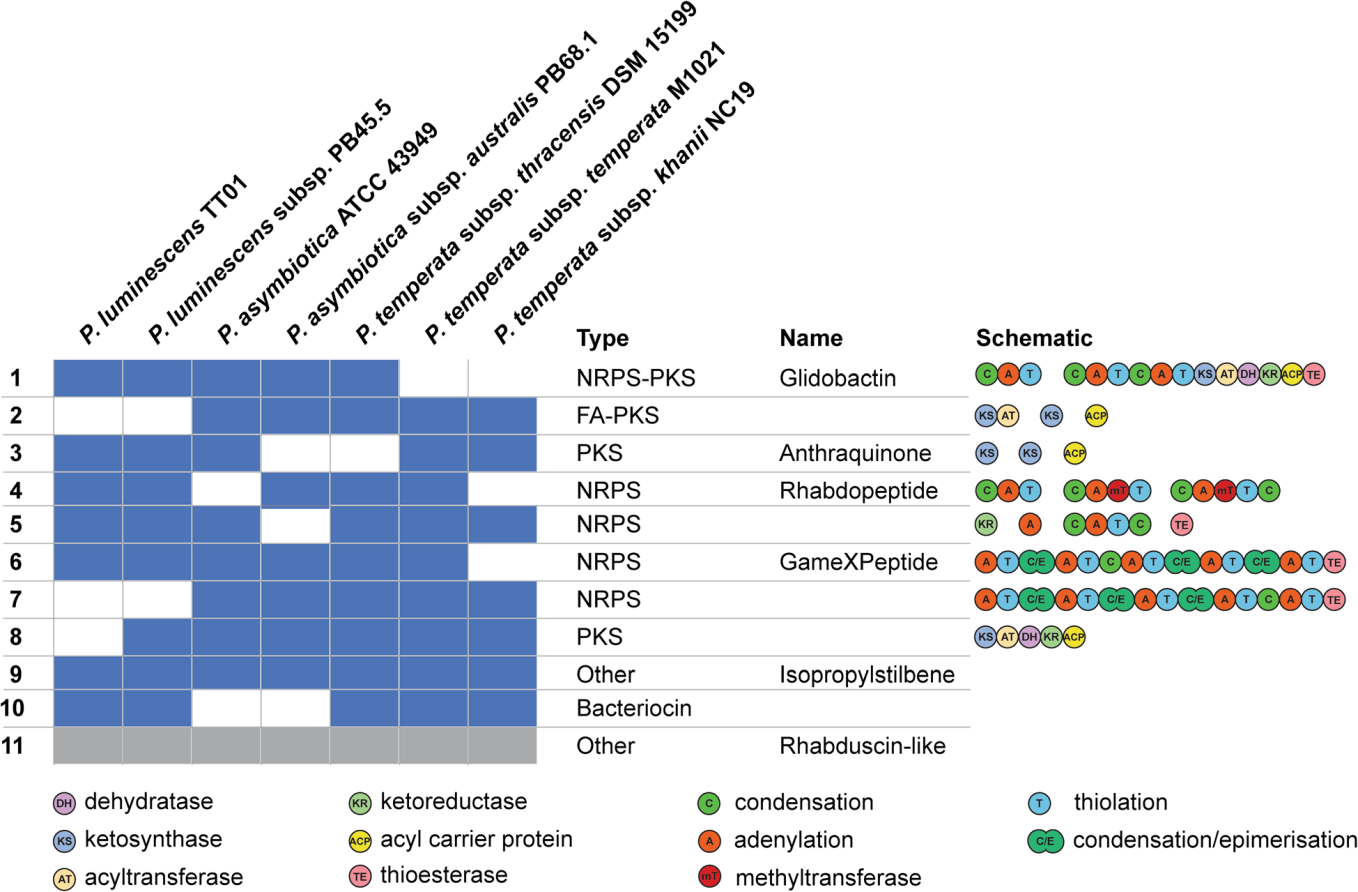
Map of highly conserved BGCs (present in at least 5 strains) in *Photorhabdus* spp. Following antiSMASH analysis, clusters were aligned using Mauve (v2.3.1) to identify homologous sequences. Domain architecture was checked using the conserved domain database from NCBI for each cluster to ensure consistency across the proposed families. Class of compound, names of identified compounds and domain structures are indicated. For all BGCs, see Additional file 5. *Grey boxes* represent the reported cluster, not identified by antiSMASH (see Methods)

The extensive core genome for the *Photorhabdus* suggests that many features of the lifestyle, regardless of the host, are conserved. One major drawback in trying to identify BGCs that are common across the genus is that the *P. temperata* NC19 and M1021 assemblies contain several BGCs that appear to be heavily fragmented. However, predicted reconstruction (see methods) of these BGC’s provides some insight into the presence or absence of clusters identified in the fully sequenced strains (Fig. 1, Additional file 5) and confirms the findings performed on the analysis of *P. temperata* NC19 by Hurst et al. [34]. A total of 75 BGCs were identified in the seven sequenced strains with several species specific BGCs. This number may however still be an over estimation of the true number of BGCs given that *P. temperata* subsp. *khanii* NC19 is one of the most heavily fragmented assemblies and contains 40 predicted clusters (some of which span whole contigs), while the average of the other members of the genus is only 21 (Table 1). It should be noted that these reconstructions may be fragmented due to rearrangements in the respective genomes as has been shown by the analysis between *P. luminescens* TTO1, *P. temperata* NC19 and *P. asymbiotica* ATCC 43949 previously [34]. Functional characterization of the BGCs by chemical analysis will be an important area of future research. Despite this, it is still interesting that there is so much apparent diversity with respect to the predicted products.

**Table 1:** Summary of *Photorhabdus* BGCs

	*P. luminescens* TTO1	*P. luminescens* subsp. PB45.5	*P. asymbiotica* ATCC 43949	*P. asymbiotica* subsp. *australis* PB68.1	*P. temperata* subsp. *thracensis* DSM 15199	*P. temperata* subsp. *temperata* M1021	*P. temperata* subsp. *khanii* NC19
NRPS	10	10	9	6	12	12	29
PKS	1	2	2	1	1	2	2
NRPS-FAS	0	1	0	0	0	0	0
NRPS-nucleoside	0	0	1	1	0	0	0
PKS-NRPS	2	3	4	3	1	0	1
FAS-PKS	0	0	1	1	1	1	1
Bacteriocin	2	1	1	2	2	2	2
Beta-lactam	1	0	0	0	1	1	1
Aminoglycoside	0	0	0	0	1	0	0
Nucleoside	0	0	0	0	0	1	0
Other	6	4	3	2	2	1	4
Total	22	21	21	16	21	20	40

*P. luminescens* TTO1 contains 23 predicted BGCs with several of the products already described, many of which have reported antimicrobial activity [17, 24, 36–44]. Ten of these BGCs correlate with a core set of secondary metabolites that exists within the genus (Fig. 1). Some of these natural products are involved in development of the nematode while strains completely deficient in secondary metabolite production fail to support nematode development (Tobias, Heinrich, Eresmann, Neubacher and Bode, unpublished results). Structural similarities, compound class comparisons and proven structure-function relationships suggests that many of these remaining products have one of two main functions; cell-cell signaling or immune evasion. We suggest that the reported antimicrobial activities of some natural products may merely be a coincidental side effect of the actual compound function similar to some antibiotics [45]. Another possibility is that the same compound might have different functions in different biological contexts as exemplified by isopropylstilbene from *Photorhabdus* acting as an antibiotic against fungi and bacteria [46], shows cytotoxic activity against insect and other eukaryotic cells [47] while also required for proper nematode development [19].

Several regions in the genomes appear to contain multiple adjacent BGCs (clusters 25, 33, 41 and 43), deduced from the presence of multiple terminal thioesterase (TE) domains that usually define the endpoint of a NRPS pathway, with three of the four present in *P. temperata* strains (Additional file 5). This may indicate a complementary function of the products of the BGC as seen for pristinamycin, a synergistically acting two-component antibiotic [48]. Identifying the products and functions of those BGCs that are species-specific (Additional files 5 and 6) may provide insights into the different niches occupied by these bacteria.

### Immune evasion mechanisms

Many of the remaining compounds have yet to have a definitive function assigned to them. However, the extensive research performed in *Xenorhabdus* and similar compounds from other species, suggests that many have immune evasion functions. There is the distinct possibility that *Photorhabdus* BGCs are essential for supporting the nematode development, perhaps helping to distinguish them from closely related species that also infect insects, without nematodal assistance, such as *Serratia* or *Yersinia* [49]. While this may be true for some compounds, we suggest that the mutualistic symbiosis has been made more successful by acquisition of new BGCs by the bacteria enabling them to more efficiently overcome the host defense and consequently, killing the host more efficiently so that both bacteria and nematode benefit.

**Fig. 2:**
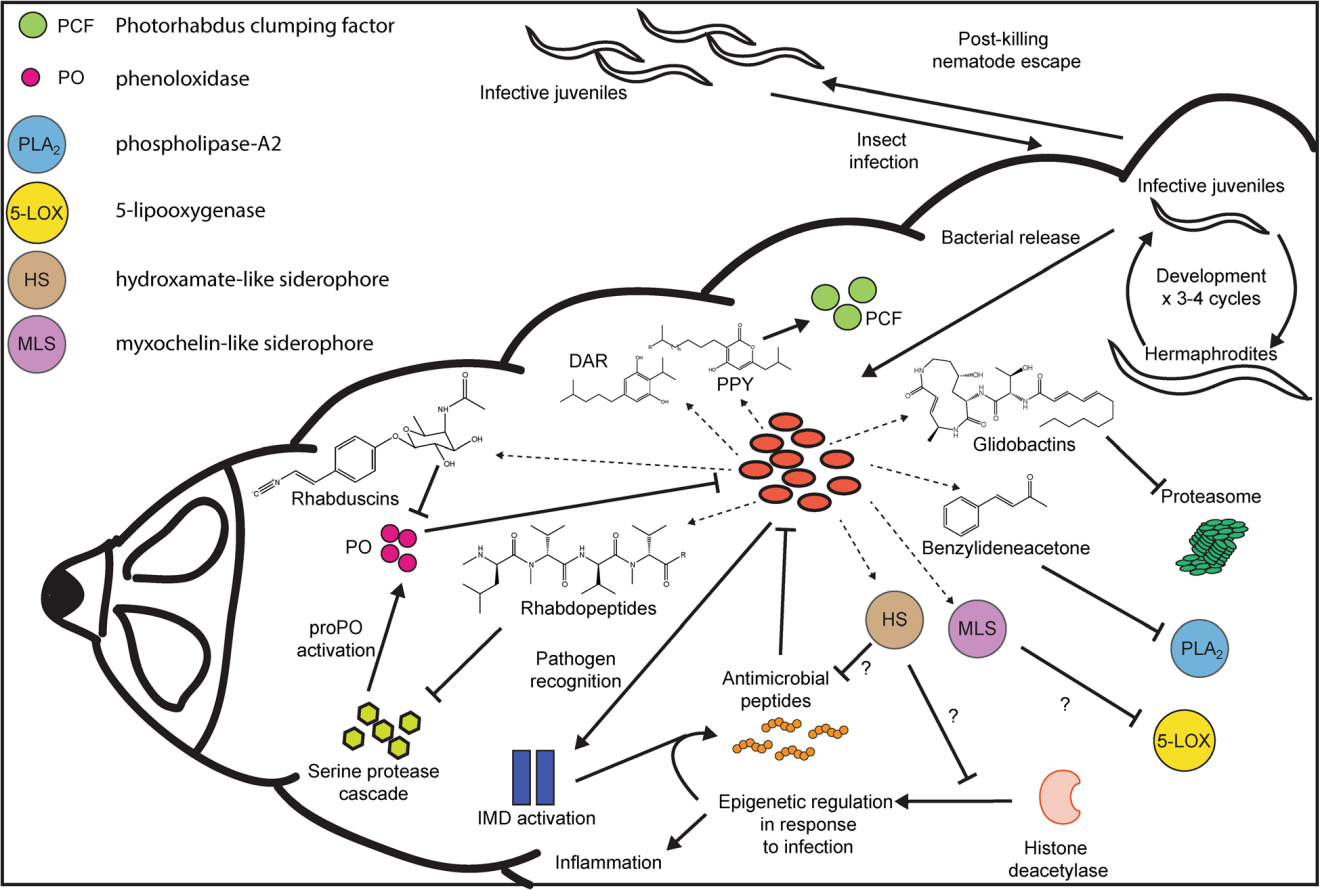
Schematic summary of the intricate tripartite lifecycle of *Photorhabdus* highlighting the produced specialized metabolites and predicted functions (indicated by a ‘?’ where unproven associations exist). Nematodes infect insects and release the bacteria inside the hemolymph before undergoing several rounds of development while the insect is killed. The bacteria release several compounds (*dashed arrows*) that variously affect the insect’s immune response. DAR = dialkylresorcinol, PPY = photopyrone

Rhabduscin is a prime example of an essential immune defensive compound produced by the IsnA and IsnB proteins, which encode an isocyanide synthase and a α-ketoglutarate dependent oxygenase respectively, together producing a potent phenoloxidase inhibitor [30, 50]. Examination of the genomes reveals that only *isnA* is present in all sequences whilst *isnB* is missing in *P. temperata* M1021 (cluster 11, Fig. 1). This suggests that instead of rhabduscin, there would be an accumulation of a reportedly unstable isocyanide-containing intermediate [50] in this strain or an alternative and yet unknown transformation of the unstable intermediate. Despite this, five species also contain the rhabdopeptide cluster (involved in mevalagmapeptide production [42]) that may be a redundant mechanism for PO inhibition (Fig. 2). Suppression of the phenoloxidase activity by rhabdopeptides has recently been described (Cai and Bode, unpublished results). This suppression method is reported to inhibit the serine protease cascade that leads to proPO cleavage. A mechanism of flexible synthesis by the rhabdopeptide system that occurs in a protein concentration dependent manner that results in differing lengths of ensuing products has also recently been uncovered (Cai, Nowak, Wesche, Bischoff, Kaiser, Fürst and Bode, submitted). This raises the possibility of a way by which the bacteria are able to either infect and suppress immune responses in a broad range of insects and efficiently evade the relevant immune systems or to address multiple targets in a single cell resulting in synergistic activity comparable to a combination therapy used in human treatments.

Siderophores are often essential in causing virulence in a range of bacteria (recently reviewed in [51–53]). One conserved cluster in *Photorhabdus* is predicted to produce a myxochelin-like siderophore (cluster 5, Fig. 1). Myxochelins have been shown to target and suppress the activity of 5-lipooxygenase [54], a key enzyme in the insect innate immune response (reviewed in [55]). Additionally, *P. luminescens* contains a further cluster with predicted siderophore function, a hydroxymate-like siderophore (cluster 74). Hydroxymate siderophores are potent histone deacetylase inhibitors. Histone deacetylases are involved in transcriptional reprogramming during wounding and infection and have been shown to repress antimicrobial peptide (AMP) production in *Galleria mellonella,* a common insect model for *Photorhabdus* virulence [56]. In addition to these specific roles, we cannot rule out the possibility that these siderophores also play a more general iron-scavenging role within the insect or nematode.

Phospholipase-2 (PLA-2) is a part of the eicosanoid biosynthesis pathway that is activated in response to recognition of pathogens by the insect. The eicosanoids are essential in mediating activation of phagocytosis and proPO production in the insect hemolymph [57]. Seo et al. (2012) have recently found that several *Photorhabdus* species are capable of inhibiting this by production of benzylideneacetone thereby preventing the recruitment of hemocytes and activation of phagocytosis [37, 58]. Benzylideneacetone is likely derived from the IPS biosynthetic pathway (extension of the phenylalanine derived cinnamoyl-CoA), which is a BGC conserved in all strains (cluster 9, Fig. 1) [38]. A further mechanism of insect immune suppression is via proteasome inhibition. Recently, glidobactin A and its iso-branched acyl derivative cepafungin, products of an NRPS-PKS hybrid gene cluster that is highly conserved (Fig. 1), were reported to be produced by *Photorhabdus* and are potent proteasome inhibitors [39, 59]. An overview of possible immune evasive and suppression mechanisms as they relate to natural products in *P. luminescens* is provided in Fig. 2.

### Two-component signal transduction systems

Six two-component systems (TCS) were conserved in all *Photorhabdus* species as well as *E. coli,* with a further system present only in all *Photorhabdus* strains. Among the conserved two-component systems is the well described CpxRA TCS, which is involved in a range of cellular processes from synthesis and translocation of cell membrane proteins [60–63] to resistance to AMPs [64] and various other virulence phenotypes [65–67]. BaeRS was also implicated in regulating multidrug resistance in *E. coli* [68] while TctED is involved in tricarboxylic acid transport [69] and UhpAB in involved in sugar transport pathways, responding to extracellular glucose [70]. The OmpR/EnvZ TCS is also well described in *E. coli* and is central in regulating the Omp locus in response to external osmolarity alterations [71, 72]. The final TCS is the PhoPQ system, which is post-translationally controlled by sRNAs [73] and responds to magnesium concentrations or AMPs in the environment [74]. However, the single TCS unique to *Photorhabdus* is the AstSR that was previously identified as being important in *Photorhabdus* phase switching [75] and identified as a likely component involved in insect infection [32].

### Cell-cell communication

We have recently reported two new classes of bacterial signaling molecules in *Photorhabdus*, namely the photopyrones (PPY) and dialkylresorcinols (DAR) [36, 40]. The DAR and PPY signaling pathways represent new methods of cell-cell communication and were discovered through the analysis of LuxR orphans (reviewed in [76]). While the DAR locus was identified in all strains (a part of the IPS biosynthesis shown in cluster 9, Fig. 1), the PPYs were only found in *P. luminescens* TTO1 and *P. temperata* subsp. *thracencis* suggesting a far less important role for PPYs (cluster 72, Additional file 5). Additionally, there are several other LuxR orphans in these bacteria with unidentified signals. One possibility is that some of the unknown clusters produce compounds can be sensed by these receptors. Another possibility that has been raised is the promiscuous activation of these receptors through compounds produced by either the nematode or insect prey, representing a form of cross-kingdom communication [41].

Only three additionally conserved regulatory proteins are present in all *Photorhabdus* examined. Two of these candidates are from the class of aforementioned LuxR orphans while the remaining is the HpaA regulator involved in the degradation of 4-hydroxyphenylacetic acid, which while absent in *E. coli* K12, is present in several other *E. coli* strains [77, 78]. It is also important to note that 872 (409 absent in *E. coli* K12) hypothetical proteins are additionally conserved with several potentially having undefined regulatory roles (Additional file 2).

### Other conserved virulence factors

Other predicted virulence factors conserved across the genus include a number of different protein toxins, a *fli* locus for flagellar assembly, a secretion system as well as various other insect associated proteins. PrtA, a protein known to be involved in insect colonization is present in all *Photorhabdus* strains [79]. Additionally, the genus contains a particularly large repertoire of protein toxins. The insecticidal toxin complex (Tc) proteins are over-represented in the total number. The Tc toxins consist of four sub-types, predicted to have different host targets [80], each of which is represented in the *P. luminescens* genomes. In total there are 16 annotated Tc protein families, all of which are present in both *P. luminescens* strains while the *P. temperata* strains have between eight and 11 proteins and the *P. asymbiotica* strains have only eight. Additionally, the repeats-in-toxin (Rtx)-like toxin are cytotoxins conserved in many Gram-negative pathogens [81] and similarly in all *Photorhabdus.* The *mcf* (makes caterpillars floppy) toxin now has an established role in insect pathogenicity in *P. luminescens* TTO1 with its presence enough to allow *E. coli* to kill insects [82–84]. Interestingly, this protein is present in all strains except for *P. temperata* M1021. However, the absence of this and a disproportionate number of other CDS that are present in all other *Photorhabdus* may just be indicative of the highly fragmented nature of this assembly in comparison to the others. Re-sequencing of this strain using long-read technology will provide more conclusive answers.

*Photorhabdus* contains only a single Type III secretion system (T3SS) that is absent in *E. coli* K12. Most strains have maintained the entire system while *P. temperata* M1021 has lost three genes (*sctC, sctV* and *sctP*), while *P. asymbiotica* and *P. temperata* subsp. *thracencis* are missing *sctE* and *sctP*, respectively. Of these missing homologs, only SctC and SctV are described as core proteins in this T3SS [85, 86] suggesting that *P. temperata* M1021 contains a non-functional T3SS. Additionally, this strain is the only strain lacking a full flagellar assembly locus (Additional file 7). Since there is significant evidence that this T3SS has a role in exporting insecticidal toxins [87], it is possible that this is merely an assembly artifact or that these bacteria instead kill insects via a different mechanism than that predicted by other *Photorhabdus.*

In terms of other host-associated proteins, each strain contains at least one predicted bacteriocin (Table 1), presumably to protect the insect cadaver from scavenging competitors. A total of five different bacteriocins were identified of which only one homolog is conserved in all *P. luminescens* and *P. temperata* strains but absent from both *P. asymbiotica* isolates (cluster 10, Fig. 1). Elucidation of the mechanism of this bacteriocin or a specific target may provide some insight into the competitors encountered by the respective species.

### Species-specific orthologs

Each of the individual species contained several coding sequences that were unique with the majority, unsurprisingly, consisting of hypothetical proteins (Table 2). However, what is interesting is that each *Photorhabdus* species appears to have a unique repertoire of regulatory proteins when compared to one another, presumably responsible for activating niche specific pathways. Within the *P. luminescens,* BLASTp searches of the non-redundant protein database show that of the regulators, one is a LysR-like regulator, one has no known domains while the four remaining are part of the XRE (xenobiotic response element) family of transcriptional regulators. The unknown regulator (plu0963/Phpb_03473) is located within the unique Tc locus indicating that it is probably a regulator for these toxins. Xenobiotics are compounds not normally found in the cell and are often detrimental. If the XRE-like regulators are in fact responding to xenobiotics and subsequently degrading them, then the elucidation of both the signal and the downstream response may provide some clear indications as to the environment in which these species are living. The *P. asymbiotica* isolates contain several additional secretion system proteins, effector molecules and what was annotated as a predicted macrophage resistance protein. This resistance protein may be a key factor in the reported ability of *P. asymbiotica* to survive and replicate within macrophages [88]. Two unique regulators from *P. asymbiotica* are the SlyA and CadC regulators (Additional files 8, 9, and 10), which have both been implicated in virulence-associated phenotypes. SlyA was found to play a role in persistence within the host cell in *Enterococcus faecalis* [89] while CadC is responsible for activating the *cadBA* locus in response to acid stress or lysine signals [90]. Perhaps more interesting however, is the absence of *cadC* in the other species. CadC is a positive regulator of the *cad* locus while negatively regulating the arginine-dependent acid response system [91]. The absence of *cadC* is a key feature of both *Shigella* and enteroinvasive *E. coli*. This absence in the other strains could indicate a form of adaptive evolution as seen in the other pathogenic enterobacteria that allows these bacteria to respond more appropriately to low pH environments.

**Table 2:** Classes of unique coding sequences in each species as identified by ortholog clustering. Full lists are available in Additional files 8, 9 and 10

CDS class	*P. luminescens*	*P. asymbiotica*	*P. temperata*
BGC associated	10	5	0
Probably virulence associated	9	10	0
Regulatory	6	5	4
Cell wall and cell processes	46	74	6
Hypothetical	57	111	22
Phage and insertion sequence	5	6	3
Total	131	211	35

## Conclusions

The identification of conserved protein families across *Photorhabdus* has helped to shed light on possible pathways essential to the intricate lifecycle of the genus. Given the roles assigned to known compounds as well as those that have yet to be confirmed but share similarities with known compounds, we suggest that many of these BGCs have been acquired as virulence factors early during speciation of the *Photorhabdus*, with one of two main functions; cell-cell communication, or modulating the insect immune response. The common belief is that many of these specialized metabolites are essential for differing antimicrobial roles. However, given the relatively low biological activity of these compounds we propose that, although they appear to have these activities, this is merely a side effect of their true function. Deconstructing the novel regulatory pathways will go a long way towards understanding each individual environment. Furthermore, the elucidation of the functions of products of the BGCs as well as whole genome comparisons to the *Xenorhabdus* species will be important areas of future research to fully understand the ecological niche occupied by these bacteria.

## Methods

### Strains and culture conditions

All *Photorhabdus* strains were grown in Luria-Bertani broth (pH 7.0) at 30 °C with shaking at 200 rpm. All strains used in this study are listed in Table 3.

**Table 3:** Strains used in this study and their accession numbers

Strain	Accession number	Reference
*Photorhabdus luminescens* TTO1	NC_005126	[2]
*Photorhabdus luminescens* subsp. PB45.5	LOIC00000000	This study
*Photorhabdus asymbiotica* ATCC 43949	NC_012962	[6]
*Photorhabdus asymbiotica* subsp. *australis* PB68.1	LOMY00000000	This study
*Photorhabdus temperata* subsp. *thracensis* DSM 15199	NZ_CP011104	[1]
*Photorhabdus temperata* subsp. *temperata* M1021	NZ_AUXQ00000000	[3]
*Photorhabdus temperata* subsp. *khanii* NC19	NZ_AYSJ00000000	[34]

### DNA methods

DNA was extracted using the DNeasy Blood & Tissue Kit (Qiagen) following the manufacturer’s instructions. *Photorhabdus* PB68.1 and PB45.5 were sequenced at Eurofins Genomics (Ebersberg, Germany) using an Illumina HiSeq2500 instrument with 150 bp paired end reads.

### Genome assembly and annotation

Raw reads were processed to trim the attached adapters and low-quality bases from both ends using Trimmomatic (v 0.32) [84] with the parameters “ILLUMINACLIP:<path to adapter sequences>:2:30:10 LEADING:3 TRAILING:3 SLIDINGWINDOW:4:15”. Further, an in-house perl script (Additional file 11) was used to discard read pairs having an average base quality less than 30, having Ns in the sequence or less than 90 bases long. After cleaning reads using the above criteria, cleaned read pairs with a minimum 90 bases in both forward and reverse reads were used for assembly. *De novo* assemblies were carried out using Velvet (v 1.2.10) [85]. To obtain optimal assemblies for both genomes, 12 assemblies for each genome were generated using odd k-mer lengths between 71 and 89, with default parameters and “-exp_cov auto”. Optimal assembly was chosen on the basis of assembled genome size, longest scaffold size, number of scaffolds, N50, N90, percentage of N in the assembly. For both genomes, the optimal assembly was obtained with a k-mer length of 89. Scaffolds longer than 300 bases were considered for gene prediction and further analyses. Following assembly, all genomes were annotated using prokka (v1.12) with default settings and --addgenes, −-compliant and --gram neg options activated [92]. Protein orthologs among the seven *Photorhabdus* strains and *E. coli* K12 were determined using proteinortho5 [93]. ANI calculations were performed using EzGenome (available at http://www.ezbiocloud.net/ezgenome). Assigning of KEGG orthology numbers and mapping to KEGG pathways was performed using the KEGG automatic annotation server [35]. QUAST was used to assess assembly quality [94].

#### Secondary metabolite cluster identification

BGCs were identified using antiSMASH v3.0 [95] together with the optional ClusterFinder algorithm using the annotated genomes as input. DNA sequences of clusters identified by antiSMASH were used in Mauve alignments to identify homologous regions to gene clusters from the already available, fully assembled genomes, enabling *in silico* reconstruction of some BGCs that were heavily fragmented. Presence of *isnA* and *isnB*, genes known to produce rhabduscin, an important immunomodulatory compound in related species, was performed manually using BLASTp (v2.2.29) as a part of the BLAST+ suite [96] with the IsnA and IsnB sequences from *Xenorhabdus nematophila* [30] used as input.

## Abbreviations

AMP, antimicrobial peptide; ANI, average nucleotide identity; BGC, biosynthetic gene cluster; DAR, dialkylresorcinol; FAS, fatty acid synthase; IJ, infective juvenile; IMD, immunodeficiency; IPS, isopropylstilbene; NRP, non-ribosomal peptide; NRPS, non-ribosomal peptide synthetase; PK, polyketide; PKS, polyketide synthase; PLA-2, phospholipase-A2; PO, phenoloxidase; PPY, photopyrone; proPO, pro-phenoloxidase; T3SS, type III secretion system; Tc, toxin complex; TCS, two-component system

## Additional files

Additional file 1: Genome assembly statistics. (DOCX 40 kb) Additional file 2: All protein ortholog families. (XLSX 394 kb) Additional file 3: KO numbers for fully assembled genomes as assigned by the KAAS server. (XLSX 262 kb) Additional file 4: KEGG pathway analysis. Numbers indicate number of CDS with matching KO numbers in each pathway. (XLSX 15 kb) Additional file 5: Complete list of BGCs identified in seven strains of *Photorhabdus*. (DOCX 1138 kb) Additional file 6: Unique BGC in each species as shown in Additional file 5. (DOCX 34 kb) Additional file 7: Coding sequences mentioned in the text and their respective locus tags. (XLSX 47 kb) Additional file 8:
*P. asymbiotica* specific coding sequences. (XLSX 20 kb) Additional file 9:
*P. luminescens* specific coding sequences. (XLSX 16 kb) Additional file 10:
*P. temperata* specific coding sequences. (XLSX 47 kb) Additional file 11: Perl code for filtering read pairs. (DOCX 14 kb)
