# Supplementary material for: Genome comparisons provide insights into the role of secondary metabolites in the pathogenic phase of the Photorhabdus life cycle
Source: BMC Genomics. 2016 Aug 3;17:537. doi: 10.1186/s12864-016-2862-4 (PMC4971723; doi:10.1186/s12864-016-2862-4)
Supplement: Additional file 1: — Genome assembly statistics. (DOCX 40 kb) [file 12864_2016_2862_MOESM1_ESM.docx]

**Additional file** **1**. Genome sequencing and assembly statistics as determined by QUAST.

| **Metric** | ***P. asymbiotica* PB68.1** | ***Photorhabdus* PB45.5** |
| --- | --- | --- |
| **Yield (Mbp)** | 1823 | 2272 |
| **Contigs (>=300)** | 209 | 106 |
| **Largest contig** | 374,072 | 493,555 |
| **Total length** | 4,918,001 | 5,425,505 |
| **GC (%)** | 42.03 | 42.70 |
| **N50** | 128,122 | 168,002 |
| **N75** | 56,764 | 97,564 |
| **Coverage** | 183.8x | 165.5x |
