# Supplementary figures and images for: Genome comparisons provide insights into the role of secondary metabolites in the pathogenic phase of the Photorhabdus life cycle
Source: BMC Genomics. 2016 Aug 3;17:537. doi: 10.1186/s12864-016-2862-4 (PMC4971723; doi:10.1186/s12864-016-2862-4)

**Additional File 5.** Complete list of BGCs identified in seven strains of *Photorhabdus.*


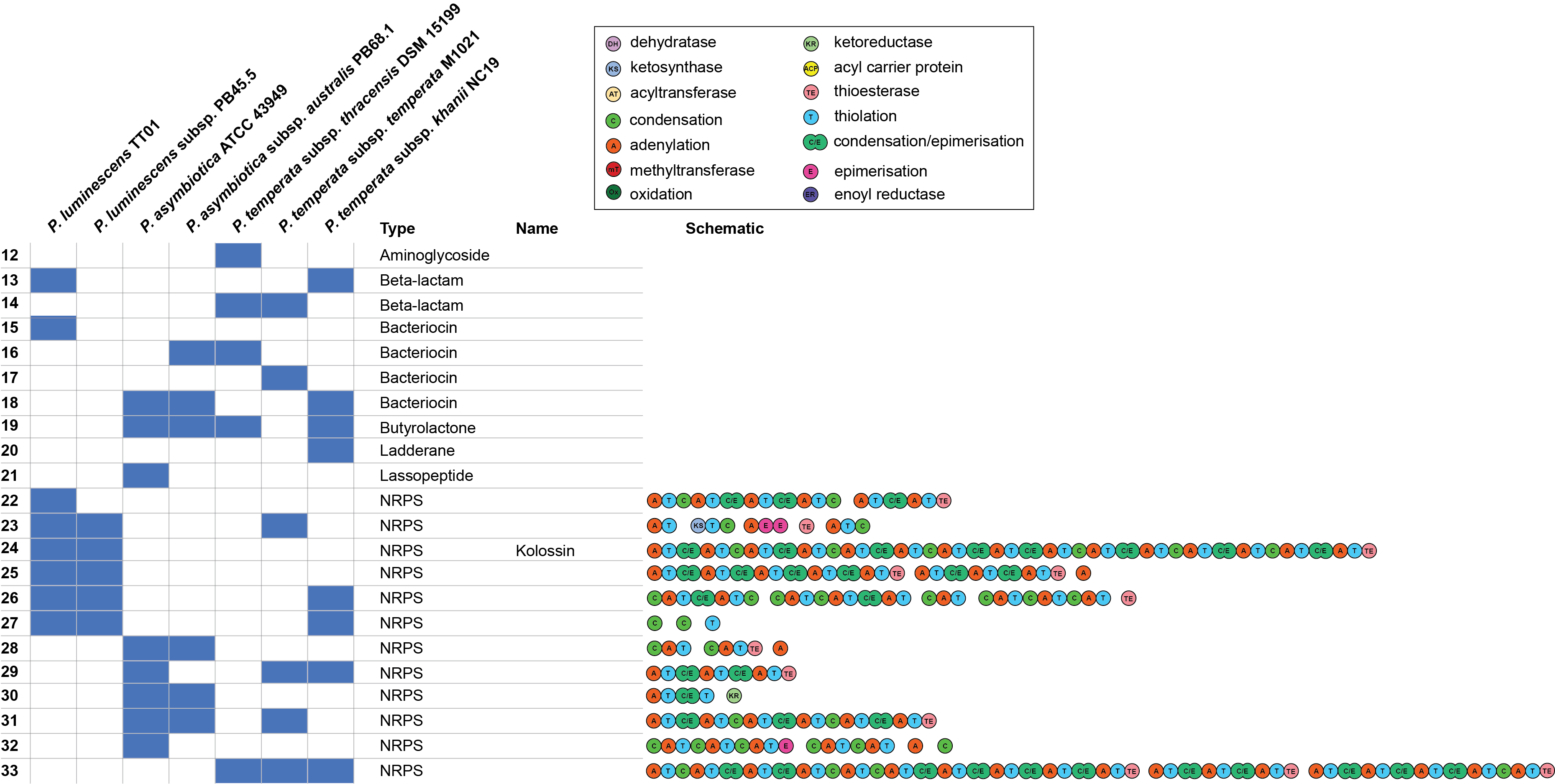


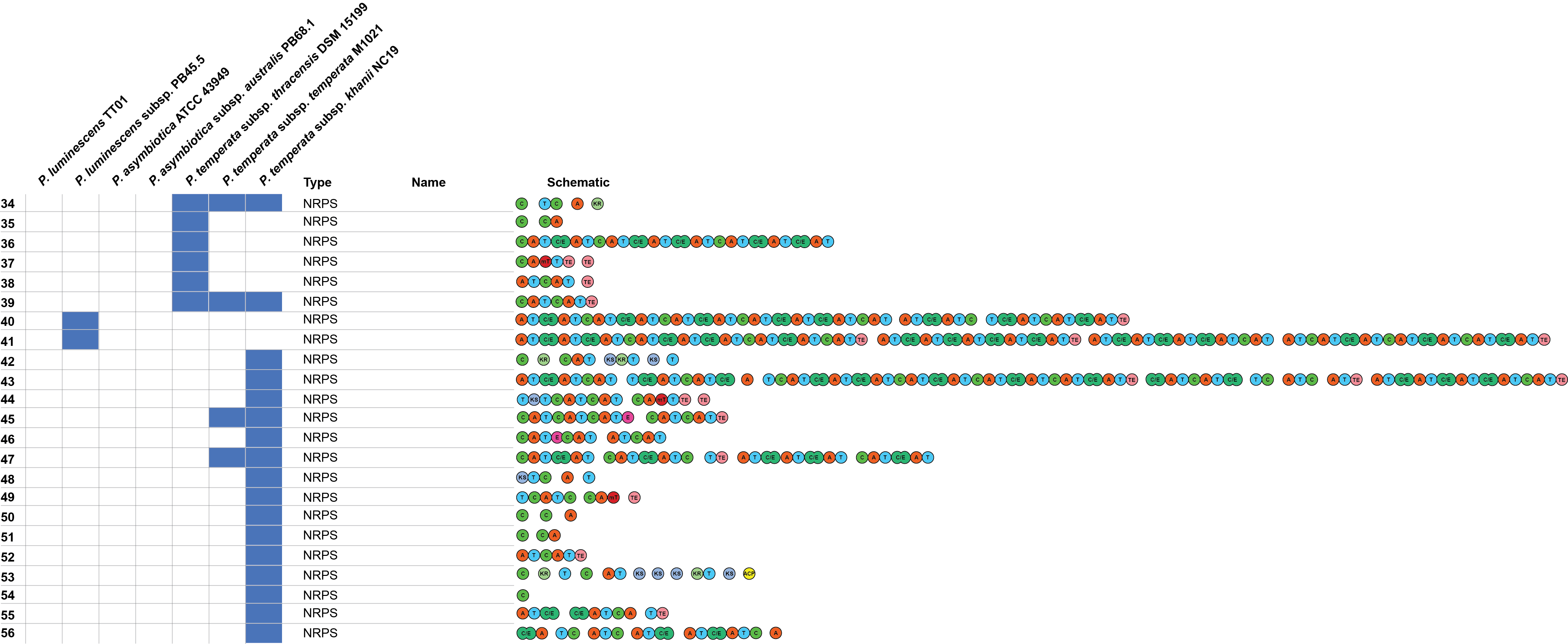

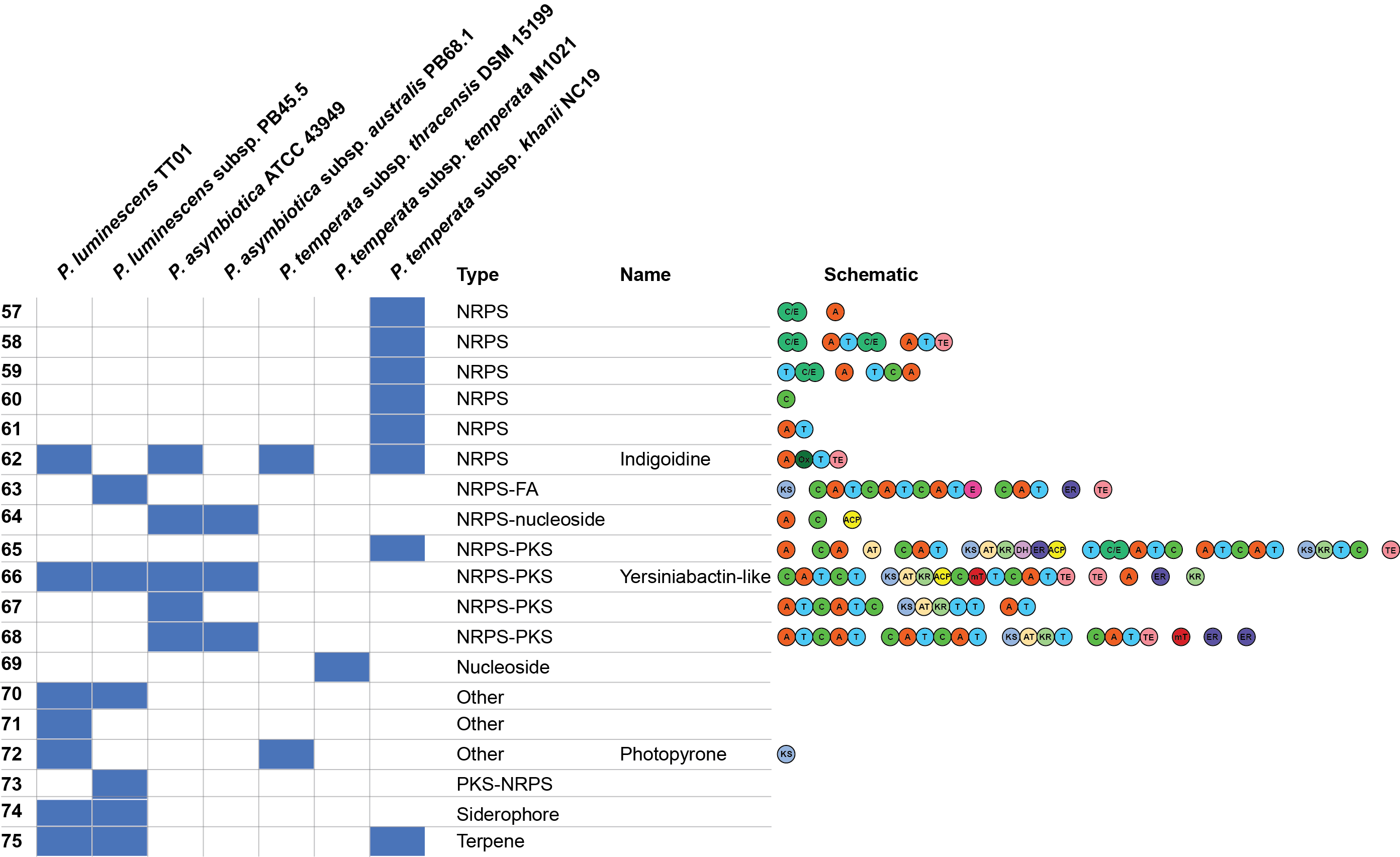

Supplement: Additional file 5: — Complete list of BGCs identified in seven strains of Photorhabdus. (DOCX 1138 kb) [file 12864_2016_2862_MOESM5_ESM.docx]
