# Supplementary material for: Genome comparisons provide insights into the role of secondary metabolites in the pathogenic phase of the Photorhabdus life cycle
Source: BMC Genomics. 2016 Aug 3;17:537. doi: 10.1186/s12864-016-2862-4 (PMC4971723; doi:10.1186/s12864-016-2862-4)
Supplement: Additional file 6: — Unique BGC in each species as shown in Additional file 5. (DOCX 34 kb) [file 12864_2016_2862_MOESM6_ESM.docx]

**Additional file 6.** Cluster numbers of those BGCs conserved in each species as shown in Additional file 5.

|  | *P. luminescens* | *P. asymbiotica* | *P. thracensis* |
| --- | --- | --- | --- |
| **Cluster** | 24 | 14 | 33 |
|  | 25 | 28 | 34 |
|  | 70 | 30 | 39 |
|  | 74 | 64 |  |
|  |  | 68 |  |
