# Supplementary material for: Genome comparisons provide insights into the role of secondary metabolites in the pathogenic phase of the Photorhabdus life cycle
Source: BMC Genomics. 2016 Aug 3;17:537. doi: 10.1186/s12864-016-2862-4 (PMC4971723; doi:10.1186/s12864-016-2862-4)
Supplement: Additional file 11: — Perl code for filtering read pairs. (DOCX 14 kb) [file 12864_2016_2862_MOESM11_ESM.docx]

################################################################################

# Usage: fastq_filter.pl <forward fastq file> <reverse fastq file> #

# #

# This script takes fastq files of both forward and reverse reads as input and outputs reads of #

# average quality >= 30 and read length >= 90 bases for both forward and reverse reads. #

# This perl script uses unix based system commands, bioawk (<https://github.com/lh3/bioawk>) #

#and bioperl (http://search.cpan.org/~cjfields/BioPerl-1.6.924/). #

# #

# author: Bagdevi Mishra. #

################################################################################

use Bio::Perl;

use Bio::SeqIO;

my $r1 = shift; #forward read file.

my $r2 = shift; #reverse read file.

chomp $r1;

chomp $r2;

$outfile1 = $r1;

$outfile1 =~ s/.fastq/_90Len30Qual.fastq/g;

$outfile2 = $r2;

$outfile2 =~ s/.fastq/_90Len30Qual.fastq/g;

my %hashR1; my %hashR2;

my @R1=bio_awk($r1);

my @R2=bio_awk($r2);

for (my $b=0; $b<=$#R1; $b++) {

chomp $R1[$b];

my @tmpR1=split(/\s+/,$R1[$b]);

my $id = $tmpR1[0];

my $qual = $tmpR1[1];

my $len = $tmpR1[2];

$hashR1{$id}= "$qual $len"; }

for (my $b=0; $b<=$#R2; $b++) {

chomp $R2[$b];

my @tmpR2=split(/\s+/,$R2[$b]);

my $id = $tmpR2[0];

my $qual = $tmpR2[1];

my $len = $tmpR2[2];

$hashR2{$id}= "$qual $len"; }

open (F,">temp.Qual30.Len_list");

for my $idsR1 (keys %hashR1) {

if ($hashR2{$idsR1}) {

my @tmp1R1=split(/\s+/,$hashR1{$idsR1});

my @tmp1R2=split(/\s+/,$hashR2{$idsR1});

if ($tmp1R1[0] >=30 && $tmp1R2[0] >= 30) {

print F "$idsR1 $tmp1R1[1] $tmp1R2[1]\n";

} } }

close F;

my @arr_list=`awk '{if (\$2 >= 90 && \$3 >= 90) print \$1}' temp.Qual30.Len_list`;

my %hash=();

foreach my $ad(@arr_list) {

my $Nval;

chomp $ad;

$Nval=`grep $ad -A1 $r1|grep -v $ad|grep -i -o "N"`;

$Nval.=`grep $ad -A1 $r2|grep -v $ad|grep -i -o "N"`;

if (!$Nval) {

$hash{$ad}=1;

} }

my $inseq1 = Bio::SeqIO->new(

-file => $r1,

-format => fastq,

);

my $inseq2 = Bio::SeqIO->new(

-file => $r2,

-format => fastq,

);

my $seq_out1 = Bio::SeqIO->new(

-file => ">$outfile1",

-format => fastq,

);

my $seq_out2 = Bio::SeqIO->new(

-file => ">$outfile2",

-format => fastq,

);

while (my $seq1 = $inseq1->next_seq) {

my $id = $seq1 ->id;

if ($hash{$id})

{

$seq_out1->write_seq($seq1);

}

}

while (my $seq2 = $inseq2->next_seq) {

my $id = $seq2 ->id;

if ($hash{$id})

{

$seq_out2->write_seq($seq2);

}

}

sub bio_awk{

$sub_a= shift;

chomp $sub_a;

`bioawk -c fastx '{ print \$name, meanqual(\$qual), length(\$seq);}' $sub_a`;

}
